# The social lottery of emotional development in medical training: Why relationships determine who develops emotional competence

**DOI:** 10.1111/medu.70199

**Published:** 2026-02-26

**Authors:** Anique E. N. Atherley

**Affiliations:** ^1^ The University of the West Indies, Cave Hill Campus Cave Hill St. Michael Barbados; ^2^ Department of Educational Development and Research, Faculty of Health, Medicine, and Life Sciences, School of Health Professions Education Maastricht University Maastricht The Netherlands

## Abstract

Equitable emotional development in undergraduate medical training doesn't happen by chance. It requires attention at three levels: supervision, educational programs, and institutional accountability. #MedEd #HealthProfessionsEd

In the aftermath of a failed resuscitation, a trainee's emotional response may be acknowledged and scaffolded … or silenced.

Becoming a doctor involves intense emotional experiences^1^ including pride, uncertainty, fear of making mistakes and stressful moments. Emotional development is the ‘desirable, sustainable enhancement of personal capacity to utilize emotional information, behaviors, and traits to facilitate desired social outcomes’.^2^ In the health professions, emotional development can be understood as the sustained growth of trainees' capacity to identify, regulate and apply emotional information (from themselves and others) in ways that support personal well‐being and reduce psychological distress,^3^ maintain professional relationships and provide optimal patient‐centred care.^1^

Although rarely named explicitly, emotional development underpins many graduate outcomes found in medical competency frameworks and curricula.^1^, ^4^ Competency frameworks worldwide^4^ require medical graduates to demonstrate empathy, professionalism, collaboration, feedback literacy, performance despite stress and effective management of interpersonal tension. These are inherently emotionally demanding outcomes.

Competency frameworks worldwide require medical graduates to demonstrate empathy, professionalism, collaboration, feedback literacy, performance despite stress and effective management of interpersonal tension.

Recent curricular innovations, including two in this issue,^1^, ^5^, ^6^ demonstrate that aspects of emotional development are teachable in structured settings.^1^ These papers also expose a quieter truth: that emotional development is built on or blocked by relationships. Access to relationships that support emotional development is shaped by hierarchy^6^ and prior and current social capital.^7^ In this commentary, I argue that emotional development in undergraduate medical training operates through two mechanisms in what can be called a social lottery: relational access and emotional legitimacy within relationships. I then end with design implications for emotional development at the level of supervisors, educational programmes and institutions.

Educational literature describes four factors for emotional development: self‐awareness, consideration of others (exhibiting empathy), connection to others (by building meaningful relationships) and capacity to influence others.^2^ The first factor, self‐awareness, can be harnessed through reflective practice, which is widely adopted in medical education.^8^ The latter three factors are underpinned by relationships. Harnessing the relational aspects of emotional development requires sociability (ability to form and maintain relationships) and intimacy.^2^ Rotation‐based clerkship models can limit sociability by threatening the ability to develop longitudinal relationships with supervisors. Beck and colleagues showed that hierarchical differences limit intimacy by reducing the visibility of trainees' emotional states by senior clinicians; this impacts trainees' expression of emotional cues.^6^ If emotional development hinges on access to supportive relationships, then the teacher–learner relationship is a potential source of inequity.

Relationships influence academic success in early undergraduate medical education^9^ and self‐regulated learning in the clinical environment.^6^ Existing literature focuses on relational access to instrumental support and resources.^7^, ^9^ However, these relationships also function as conduits for less explicit forms of support such as emotional scaffolding and buffering stress.^6^ The stress‐supervision paper in this issue illustrated how emotionally attuned supervision can transform a stressful situation into a learning opportunity.^6^ Yet, existing literature suggests that some trainees are left to manage their emotional responses alone.^7^, ^10^ When supervisory relationships are informal and unevenly distributed, access to emotionally supportive relationships becomes dependent on social positioning, and not educational design. To understand why access to emotionally supportive relationships is uneven, it is necessary to examine how relationships are formed in clinical learning environments.

When supervisory relationships are informal and unevenly distributed, access to emotionally supportive relationships becomes dependent on social positioning and not educational design.

Relationships between medical students, junior doctors and attendings emerge through well‐described social processes: serendipity (occurrence of *chance encounters*), homophily (*tendency to connect with others similar to oneself*) and propinquity (*social or geographical proximity increasing likelihood of interaction*).^9^ Evidence from the United Kingdom shows that under‐represented students struggle at relationship‐building and may not recognise the benefits of relationships, which are implicit and delayed.^11^ Serendipity, homophily and propinquity are mechanisms that systematically advantage some trainees over others in gaining access to emotionally supportive relationships. Importantly, inequity in emotional development opportunities is not shaped only by *who* trainees gain access to, but *what* is recognised as legitimate within those relationships.

Emotional legitimacy considers whether emotions are acknowledged by clinical insiders as a valid input for learning and are not dismissed as a distraction.^12^ In environments where emotional expression is devalued, even relationships may still fail to support emotional development.^5^, ^13^, ^14^ Therefore, inequitable emotional development arises not only from unequal relational access but from unequal access to relationships that are emotionally legitimate. In medical education, empathy has historically been framed as a predominantly cognitive skill. This lens may unintentionally normalise emotional distancing as professional behaviour.^5^, ^13^ Even though emotions are known to be integral to clinical tasks and learning,^14^ medicine continues to privilege a flavour of empathy that is void of emotion.^13^ When emotions are rendered invisible, learning opportunities can be missed and supervisory relationships can fail to scaffold transformative learning.^6^, ^14^ Trainees who are more isolated may be doubly disadvantaged: first by reduced access to supportive relationships^11^ and second by relational contexts where emotional expression is discouraged.^13^

Even though emotions are known to be integral to clinical tasks and learning, medicine continues to privilege a flavour of empathy that is void of emotion.

Designing undergraduate medical training for equitable emotional development requires attention at three levels: supervision, educational programmes and institutional accountability:

Designing undergraduate medical training for equitable emotional development requires attention at three levels: supervision, educational programmes and institutional accountability.

- **Supervision:** Emotional scaffolding^6^ should be an explicit supervisory expectation. Leveraging faculty development programming and using trainee feedback to evaluate supervisors on both teaching *and* emotional competence could identify emotionally supportive supervisors.
- **Educational programme:** proactively offering under‐represented trainees opt‐in access to proven, emotionally supportive mentors, advisors or coaches. Additionally, structured moments where emotional responses to clinical experiences are acknowledged should be curated; existing emotional skills interventions^1^, ^5^ operate at this level. Leveraging the power of peer relationships and the significant emotional support they provide^7^ could also be helpful through intentional peer debriefs.
- **Institution:** align emotional development expectations with competency frameworks that govern the training programme by linking emotional scaffolding to entrustable professional activities (EPAs) and graduate outcome frameworks.^10^

Emotional development cannot be nurtured through curriculum alone; it requires intentional design considering the relational architecture of training, rather than being left to chance.

Emotional development cannot be nurtured through curriculum alone; it requires intentional design considering the relational architecture of training, rather than being left to chance.

## AUTHOR CONTRIBUTIONS

**Anique E. N. Atherley:** Writing—original draft; writing—review and editing.

## CONFLICT OF INTEREST STATEMENT

I have no conflicts of interest.

## Data Availability

Data sharing is not applicable to this article as no datasets were generated or analysed during the current study.
